# Neutralization of tier-2 viruses and epitope profiling of plasma antibodies from subtype-C HIV-1 infected north Indians; implications for MPER directed HIV-1 neutralization

**DOI:** 10.1186/1471-2334-12-S1-O19

**Published:** 2012-05-04

**Authors:** Raiees Andrabi, Manju Bala, Rajesh Kumar, Anjali Hazarika, Kalpana Luthra

**Affiliations:** 1 All India Institute of Medical Sciences, Ansari Nagar, New Delhi, India; 2 Regional STD Teaching Training & Research Centre, Safdarjang Hospital, New Delhi, India

## Background

Evaluation of the broad and potent neutralizing antibody (bNAb) responses in sera of HIV-1 infected individuals is important for immunogen design.

## Methods

Plasma of eighty naïve HIV-1 seropositive Indian patients were tested for neutralization against a panel of 3 subtype-B and 5 subtype-C tier 1 and tier 2 viruses. Three plasma, found to be broadly neutralizing (bNP), were mapped with a set of 212 consensus-C gp160 overlapping peptides (15mer each). 50% binding titers were determined by ELISA using consensus-C V3 (35mer), IDR loop (19mer) and MPER (24mer) peptides.

## Results

The patients (30 females and 50 males; age range: 20-57 years) had been infected for different time periods (few days to seven years). The viral load ranged from 156-2180000 RNA copies/ml; mean CD4 count was 346 cells/mm^3^ while mean total IgG levels was 12.3 mg/ml. 64 plasma (80%) neutralized at least one virus while 16 (20%) did not show any neutralization. Three plasma samples AIIMS206, AIIMS239 and AIIMS249 neutralized all eight viruses tested. Subtype-specific neutralization predominated, plasma antibodies being more effective against subtype-C than subtype-B viruses (p=0.004). Epitope specificities of the three bNP mapped to V2, V3 and C5 of gp120 and ID region, MPER and CT of gp41. Depletion and competition with V3, MPER and IDR peptides showed modest effect on neutralization except for AIIMS239 which showed dependence on MPER directed antibodies with most viruses.

## Conclusions

Reactivity to the highly immunogenic V3 region showed dependence on plasma viremia. Anti-MPER antibodies may play a crucial role in HIV-1 clade C viral neutralization.

